# Variation of the uterine flexion angle before and after voiding in nulliparous women with anteverted uterus: FLEXIMIX study

**DOI:** 10.1186/s12905-026-04778-4

**Published:** 2026-08-19

**Authors:** Benoît Chiron, Paul Aujoulat, Lisa Gergaud, Camille Fouwé, Louise Hétuin, Mélissa Lotton, Anaëlle Le Hars, Audrey Le Goff-Coquet, Antoine Dany

**Affiliations:** 1 Department of General Practice, University of Brest, 22, av. Camille Desmoulins, F-29200 Brest, France; 2 Univ Brest, UR 7479 SPURBO, Brest, FR-29200 France

**Keywords:** Bladder filling, Intrauterine device insertion, Nulliparous, Transabdominal ultrasound, Uterine flexion angle

## Abstract

**Background:**

Resistance during intrauterine device insertion (IUD) may be influenced by the uterine flexion angle. While bladder filling has been used to facilitate catheter insertion for hysteroscopy and embryo transfer, the only study assessing this strategy for IUD insertion found no benefit. However, the physiological effect of bladder filling on uterine alignment has not been rigorously evaluated. This effect may be particularly relevant in nulliparous women with anteverted uteri. The study aimed to evaluate the variation in uterine flexion angle between full and empty bladder conditions in nulliparous women, and to determine the prevalence of uterine anteversion in a European population.

**Methods:**

Prospective single-center study included 250 nulliparous women aged 18 years or older. Participant underwent two transabdominal ultrasounds: with a full bladder / after voiding. Uterine version and flexion were assessed by measuring the angles formed by the uterine body, cervix, and vaginal axis.

**Results:**

Among the 215 participants with uterine anteversion (87.0%), paired flexion angle measurements were available for 212 (84.8% of the total cohort). Among these 212 women, the mean change in flexion angle was 48.0° (SD 36.7°, *p* < 0.001; Cohen’s d = 1.31). In 60.8% of cases, bladder filling changed an acute flexion angle to an obtuse one. The point of uterine flexion was located approximately 20–21 mm from the external cervical os, just beyond the internal os, suggesting a mechanical effect of bladder distension on uterine alignment.

**Conclusions:**

Bladder filling significantly modifies uterine flexion in nulliparous women with anteversion.

This physiological effect provides a plausible anatomical mechanism that may facilitate IUD insertion. Further interventional studies are needed to determine its clinical applicability.

**Trial registration:**

ClinicalTrials.gov, trial registration number: NCT06669845, registration date: 2024-10-31.

## Background

The intrauterine device (IUD) accounts for 16.8% of contraceptive methods used worldwide [1]. Its insertion is often painful for patients and may be technically challenging for operators. Insertion difficulties most commonly occur with the entry into the uterine cavity [2, 3]. To facilitate insertion, a tenaculum is often used, but is associated with increased pain [4].

In clinical practice, insertion appears to be easier when the bladder is full, although no scientific evidence supports this impression. In in vitro fertilization procedures, a full bladder improves embryo transfer outcomes by reducing pain and failure rates [5]. For hysteroscopy, a full bladder reduces pain, procedure time and increases first-attempt success rates, without impairing patient acceptability [6].

For IUD insertion, only one randomized trial has assessed the effect of bladder filling, without demonstrating any benefit [7]. In accordance with common insertion practice, tenaculum has probably be used systematically from the start of the insertion procedures, potentially masking any benefit of a full bladder on pain or ease of insertion.

Theoretically, the obstacle posed by uterine anteflexion during IUD insertion should diminish once the uterus straightens beyond a right angle (> 90°). The only available study reporting an increase in flexion angle with bladder filling including 21 women with uterine anteversion and tended to support this hypothesis [8]. Scarce data report a uterine anteversion prevalence of 87%, assessed by transabdominal ultrasound in non-European populations [9].

The primary objective of this study was to determine the variation in uterine flexion angle before and after voiding in nulliparous women with uterine anteversion.

Secondary objectives were to 1/ determine the proportion of nulliparous women whose anteflexion angle changed from obtuse to acute after voiding; 2/ estimate the prevalence of anteversion in nulliparous women; and 3/ measure the mean and distribution of the distance between the external cervical os and the uterine flexion point under both full and empty bladder conditions.

## Methods

### Study design

This was a single-center, cross-sectional clinical study comparing the uterine anteflexion angle before and after voiding, using transabdominal pelvic ultrasound. All STROBE guideline items were followed to ensure the methodological quality.

### Population

Inclusion criteria included nulliparous women aged 18 or older and reporting the need to urinate. Exclusion criteria were: current or previous pregnancy, history of elective or therapeutic abortion, hysterectomy, cervical conization, primary amenorrhea, or an urgent need to void.

### Recruitment and inclusion

Participants were recruited through posters, email, and direct approach of students, conducted both before and during inclusion period, at the University and the University Hospital of Brest, France. Inclusions took place over two weeks in November and December 2024.

### Ultrasound protocol

Two general practitioners with expertise in pelvic ultrasound acquired the images, and a third examiner independently reviewed all of them. Examinations were conducted using two Sonoscanner^®^ ultrasound devices with identical settings and identical low-frequency curved-array probes.

To ensure measurement consistency, the two sonographers underwent an initial consensus training during the first ten study examinations. During this period, they alternately acquired the ultrasound images and jointly reviewed the images to harmonize the ultrasound plane, probe pressure, anatomical landmarks, and measurement procedures. All subsequent examinations were then performed according to this standardized approach.

To ensure participant comfort, the first ultrasound examination was performed after they reported a need to void but before reaching an urgent need to void, in accordance with the predefined exclusion criteria.

After confirming eligibility and obtaining written informed consent, participants underwent a first transabdominal pelvic ultrasound in the supine position with a full bladder. The probe was angled to capture a parasagittal view along the main longitudinal axes of the uterus, cervix and vagina. The three corresponding reference lines were then drawn on this image. The ultrasound system automatically calculated the angles of uterine version and flexion. Cervical length was measured from the external cervical os to the flexion angle.

In the absence of standardized thresholds in the literature, reproducible classification criteria were defined. Uterine version was categorized according to the angle between the longitudinal axis of the uterine body and the vaginal axis as follows: marked anteversion (< 150°), moderate version (150–180°), and retroversion (> 180°). Uterine flexion was the angle between the axes of the cervix and uterine body.

After voiding, a second ultrasound was performed using the same imaging protocol and measurements. All images were de-identified.

### Collected variables and endpoints

Participant data collected included age, date of last menstrual period, presence of an IUD, and history of abdominal, urological, or gynecological surgery. Ultrasound data included the values of the uterine flexion and version angles, and the distance from the external cervical os to the uterine flexion angle, both before and after voiding.

The primary outcome was the difference in mean uterine flexion angle between full and empty bladder states among nulliparous women with an anteverted uterus (marked or moderate).

Secondary outcomes were: 1/ the proportion of women whose uterine flexion angle changed from acute (< 90°) with an empty bladder to obtuse (> 90°) with a full bladder; 2/ the prevalence of uterine anteversion as assessed with an empty bladder; 3/ the mean and distribution of the distance between the external cervical os and the uterine flexion angle with a full and with an empty bladder.

### Sample size calculation

The literature reports a prevalence of uterine anteversion ranging from 80% to 90% [9, 10]. A conservative estimate of 80% was used for sample size calculation. Assuming a two-tailed alpha risk of 5% and a moderate effect size (Cohen’s d = 0.50), 200 paired observations would yield a statistical power greater than 99% to detect a difference in the anteflexion angle between the full and the empty bladder conditions. With 250 participants, the 95% confidence interval for the estimated prevalence of uterine anteversion was expected to have a margin of error less than 5%.

### Statistical analysis

Descriptive statistics included proportions with 95% confidence intervals. For comparative analysis, each full bladder measurement was compared to its corresponding empty bladder value.

Normality of distributions was assessed using the Shapiro–Wilk test. Paired one-tailed t-tests were used for normally distributed data and Wilcoxon signed-rank tests otherwise. One-tailed tests were prespecified in the statistical analysis plan because the study hypothesis was directional, namely that bladder filling would increase the uterine flexion angle. A decrease in the angle was considered not clinically relevant to the study objective, in accordance with the ICH E9 statistical principles for clinical trials. Participants with unmeasurable angles with an empty bladder were excluded from the analysis and considered as non-anteverted in intention-to-treat analyses.

All analyses were performed using R software, version 4.5.1 (R Core Team, 2023).

### Ethical approval and consent

All participants received detailed information regarding risks and constraints and provided written informed consent. The study protocol was approved by an Ethics Committee for the Protection of Persons.

## Results

### Participant characteristics

The 250 included women ranged in age from 18 to 49 years, with a mean age of 21.9 years (SD 3.8). In total, 17.6% (95% CI: 12.9–22.3) reported current IUD use, of which 77.3% were copper IUDs and 22.7% were hormonal IUDs. A history of abdominal, gynecological, or urological surgery was reported by 5.6% (95% CI: 2.7–8.4) of participants (Table 1).

**Table 1:** Participant characteristics

Participant Characteristics (*N* = 250)	Count or Mean	% or SD
IUD use	44	17.6%
Age (years)	21.9	± 3.8
Copper IUD	34	77.3%
Hormonal IUD	10	22.7%
Medical history (abdominal, urologic, gynecologic)		
Yes	14	5.6%
No	236	94.4%
Uterine version with an empty bladder (*n* = 247*)		
Total anteversion (< 180°)	215	87.0%
* Marked anteversion (< 150°)*	*194*	*78.5%*
* Moderate anteversion (150–180°)*	*21*	*8.5%*
Retroversion (> 180°)	32	13.0%

*n = 247: Uterine version could not be measured in 3 participants

### Variation in uterine flexion angle in women with anteverted uteri

The variation in uterine flexion angle could not be calculated in 6 participants because flexion angle measurements were missing at either the empty bladder condition (*n* = 3) or the full bladder condition (*n* = 3). Among the remaining 244 interpretable cases, 212 (84.8%) women had anteverted uteri and were included in the primary analysis. As both full and empty bladder flexion angles showed non-normal distributions (Shapiro–Wilk test, *p* < 0.01), paired comparisons were performed using the Wilcoxon signed-rank test. The mean flexion angle was significantly higher with a full bladder compared to an empty bladder (130.9° vs. 82.9°, *p* < 0.001), corresponding to a mean increase of 48.0°. The effect size was large (Cohen’s d = 1.31), indicating a marked change in uterine flexion with bladder filling (Table 2).

**Table 2:** Cervical length and uterine flexion angles with empty and full bladder

	Empty bladder	Full bladder	Mean difference	Effect size / *p*
Cervical length (mm) median [Q1-Q3]	19.6 [17.8–22.0]	20.9 [18.9–23.4]	1.3	d = 0.27
Flexion Angle				
All Anteverted Uteri (212)	82.9°	130.9°	**48.0°**	d = 1.31 / < 0.001
* Marked Anteversion (191)*	73.9°	128.0°	54.1°	< 0.001
* Moderate Anteversion (21)*	164.6°	156.4°	-8.2°	0.9
Retroversion (32)	213.4°	179.4°	-34.0°	< 0.001
* Unmeasurable case (6)*	*NA*	*NA*	*NA*	*NA*

The mean change in flexion angle was + 54.1° in women with marked anteversion (*n* = 191), − 8.2° in those with moderate anteversion (*n* = 21), and − 33.9° in those with retroverted uteri (*n* = 32). Post hoc pairwise comparisons between these three groups (Dunn’s test for multiple comparisons with Holm correction) confirmed statistically significant differences between the marked anteversion and moderate anteversion groups (*p* < 0.001), as well as between the “anteversion” and “retroversion” groups (*p* < 0.001).

### Change in flexion angle (Acute to obtuse)

**Fig. 1:**
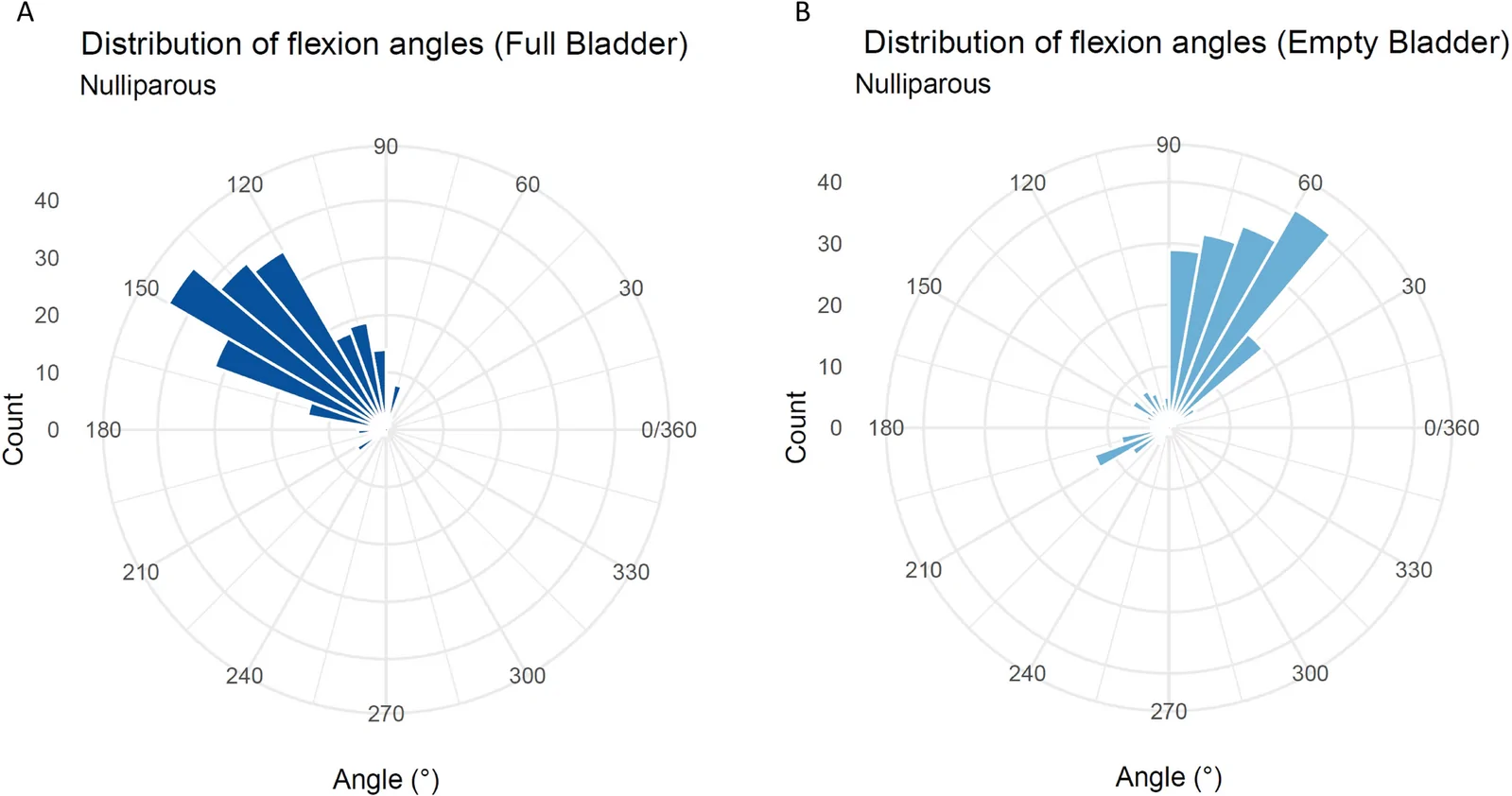
Polar histograms illustrating the distributions of flexion angles under full and empty bladder conditions

Among all women (*n* = 250), a change from an acute flexion angle with an empty bladder to an obtuse angle with a full bladder was showed for 60.8% (95% CI: 54.7–66.8). For the others, 5.2% (95% CI: 2.4–7.9) remained acute, 31.6% (95% CI: 25.8–37.3) remained obtuse, and 2.4% (95% CI: 0.5–4.3) had angle variation that could not be measured. Comparison of empty and full bladder conditions revealed a clear modification in flexion angle distribution, characterized by a shift from predominantly acute angles to more obtuse with bladder filling (Fig. 1).

In the target population of women with anteverted uteri (*n* = 212), the proportion of change from acute to obtuse was 69.8% (95% CI: 63.6–75.9) (Table 3).

**Table 3:** Distribution of uterine flexion angle categories with empty and full bladder

Empty Bladder	Full Bladder	%	(95% CI)
Total sample (*N* = 250)			
Acute	Acute	5.2	2.4–7.9
Acute	Obtuse	**60.8**	54.7–66.8
Obtuse	Obtuse	31.6	25.8–37.3
*Unmeasurable*		*2.4*	*0.5–4.3*
Anteverted uteri (*N* = 215)			
Acute	Acute	6.1	2.9–9.2
Acute	Obtuse	**69.8**	63.6–75.9
Obtuse	Obtuse	22.8	17.2–28.4
*Not measurable*		*1.4*	*0-2.9*

### Prevalence of uterine anteversion

The uterine version angle could not be measured with an empty bladder in 3 participants. Among the remaining 247 women, 78.5% had marked anteversion (angle < 150°), 8.5% had moderate anteversion (150–180°), and 13.0% had retroversion (angle > 180°) (Table 1).

### Distance from external os to flexion angle

The distribution of the distance from the external cervical os to the uterine flexion angle was slightly skewed but close to normal. With a full bladder, the mean distance was 21.4 mm (SD 3.9), and the median was 20.9 mm [18.9–23.4]. With an empty bladder, the mean was 20.3 mm (SD 3.5), and the median was 19.6 mm [17.8–22.0]. The effect size was small (Cohen’s d = 0.27), suggesting a negligible difference between full and empty bladder conditions (Table 2).

## Discussion

### Main findings

Among nulliparous women with uterine anteversion, the mean variation in the uterine anteflexion angle between the empty and full bladder states was 48.0°, a statistically significant change (*p* < 0.001) with a large effect size (Cohen’s d = 1.31). Among the 250 women included, 60.8% showed a transition from an acute flexion angle with an empty bladder to an obtuse angle with a full bladder. The prevalence of uterine anteversion in this population was 87.0%. Finally, assuming that resistance to IUD insertion occurs at the point of uterine flexion, the estimated location of this angulation was 20 to 21 mm from the external cervical os, which is consistent with the clinical experience of clinicians who do not routinely use a tenaculum.

### Comparison with the literature

#### Bladder influence

Classical anatomical treatises based on cadaveric dissections without a full bladder, primarily describe the characteristics and prevalence of uterine version and flexion angles. The potential influence that the filling of one pelvic organ may exert on the position of another has been briefly mentioned [11, 12]. More recent literature provides limited data on the influence of bladder filling on uterine angle variation [9, 13]. A 2020 study investigated ways to improve pelvic MRI quality by modifying certain parameters, including bladder filling [8]. Flexion angles were compared in 26 women across three levels of bladder fullness. In the subgroup of 21 women with anteverted uteri, mean flexion angles were 115°, 134°, and 145°, respectively with an empty, moderately filled, and full bladder. The mean age of the sample was 36 years, suggesting that most participants were likely multiparous.

#### Prevalence of anteversion/retroversion

Uterine anteversion is sometimes considered the angle between the cervix and the vaginal axes. Using this definition, its prevalence among nulliparous women was estimated at 80.4% in a 2014 study [10]. An Australian study compared the prevalence of uterine version assessed by transabdominal ultrasound without prior instruction, and by transvaginal ultrasound after voiding [9]. Among nulliparous women, the prevalence of retroversion was 7.6% using transabdominal ultrasound and 14.3% using transvaginal ultrasound with an empty bladder. Bladder filling thus appeared to correct retroverted uteri.

However, the authors’ conclusion that transvaginal ultrasound should be considered as the reference method for assessing uterine version is debatable. In our study, the prevalence of retroversion by transabdominal ultrasound was 13.0%, very close to the 14.3% reported using transvaginal ultrasound after voiding. This suggests that bladder emptying, rather than the imaging modality, may be the primary factor influencing uterine version assessment.

#### Cervical length

In our study, the distance between the external cervical os and the point of uterine flexion averaged 20 to 21 mm. Given that the cervix is considered a rigid structure, flexion occurs just beyond the internal os, at the level of the uterine isthmus.

The literature traditionally defines cervical length as the distance between the external and internal os, but does not report data on the distance from the external os to the point of flexion. Studies conducted during the second trimester of pregnancy report cervical lengths ranging from 25 to 35 mm [14]. The difference may be related to structural changes of the uterus induced by the gravid state [15].

During hysteroscopies and IUD insertions, difficulties in advancing instruments typically occur at similar depths, suggesting that uterine flexion, rather than resistance from the cervix itself, is the main cause of obstructions.

### Strengths and limitations

One of the main strengths of this study lies in its novelty. To our knowledge, no previous study has specifically and rigorously examined variation in the uterine flexion angle according to bladder filling status. The sample consisted exclusively of healthy nulliparous women, whose mean age and standard deviation suggest that most had not undergone prior obstetric or gynecologic care. Before inclusion, participants were unaware of their uterine version status and only the endpoint of the variation of their flexion angle was disclosed to them. These elements minimize the risk of self-selection bias and enhance the external validity of the estimated prevalence of anteflexion, making it generalizable to a comparable population.

The use of transabdominal rather than transvaginal ultrasound facilitated participation by avoiding an invasive procedure that could have limited recruitment of nulliparous women. This approach, well suited for visualizing the uterus, enabled reliable measurement of axes and lengths without the interference of pressure exerted by a vaginal probe. Nonetheless, in some cases, uterine visualization was impeded by bowel gas artifacts, and cervical measurements were occasionally obscured by the brightness of a copper IUD. Additionally, the terminal axis of the vagina often appeared curved, requiring a visual estimation of its long axis.

Although no formal assessment of interobserver or intraobserver reproducibility was prespecified, measurement consistency was enhanced through an initial consensus training of the two sonographers during the first ten study examinations and by independent review of all 250 image pairs by a third examiner.

The thresholds used to categorize uterine version and flexion angles were based on arbitrary values due to the lack of consensus in the literature, which limits comparability with studies using different criteria.

Another methodological limitation concerns the subjective nature of the inclusion criterion based on the participant’s sensation of bladder fullness. The absence of bladder volume measurement may have resulted in significant interindividual variability at the time of the first ultrasound, potentially affecting the observed magnitude of angular variation. This exposure heterogeneity may explain certain individual differences, particularly cases with minimal angle variation due to insufficient bladder filling.

Six examinations could not be used to determine angle variation due to insufficient image quality. Even assuming these exclusions all concerned retroverted uteri, the estimated prevalence of retroversion would be only marginally affected (from 12.96% to 12.80%), supporting the robustness of the findings.

A final source of uncertainty concerns the best way to define uterine version: should it be based on the axis of the cervix or of the uterine body relative to the vagina? This unresolved question warrants caution when interpreting version categories.

### Perspectives

In this study, considerable heterogeneity in bladder filling volumes was observed during ultrasound. As the protocol did not include volume measurement, no correlation could be drawn between bladder volume and uterine flexion angle, although such analysis would have been relevant. This methodological choice was intended to test two hypotheses in a simple and practical way: the validity of the concept of angle opening induced by bladder filling, and its direct clinical applicability through a straightforward instruction for patients to attend IUD placement appointments with a full bladder.

Several questions now arise. What bladder volume is required to achieve sufficient opening of the flexion angle? To what extent might this strategy reduce the need for tenaculum use during IUD insertion? Does the improved comfort gained during insertion outweigh the discomfort of a full bladder, particularly in the event of appointment delays? If so, what hydration protocol would ensure both an adequate bladder volume and a tolerable need to void?

In parous women, does gestation reduce uterine flexibility leading to a less pronounced flexion angle, which contribute to easier IUD insertion than in nulliparous? We are currently conducting further studies to explore these questions. Overall, this study highlights a physiological mechanism with potential clinical utility. However, the present study was designed to investigate a physiological mechanism rather than a clinical intervention. Whether this anatomical change translates into easier, less painful, or more successful IUD insertion remains to be demonstrated in future interventional studies.

## Data Availability

Data is available upon request to the corresponding author.

## Abbreviations

IUD: Intrauterine device
